# Urinary plasminogen as an early marker of diabetic kidney disease in children with type 1 diabetes mellitus: a cross-sectional study

**DOI:** 10.1007/s00431-025-06278-3

**Published:** 2025-06-27

**Authors:** Mihriban İnözü, Gönül Büyükyılmaz, Begüm Avcı, İsmail Selçuk Aygar, Fatma Semsa Çaycı, Seyit Ahmet Uçaktürk, Eda Mengen, Sare Gülfem Özlü, Umut Selda Bayrakçı

**Affiliations:** 1 Department of Pediatric Nephrology, Ankara City Hospital, Ankara, Turkey; 2 Faculty of Medicine, Department of Pediatric Nephrology, Ankara Yıldırım Beyazıt University, Ankara, Turkey; 3 Department of Pediatric Endocrinology, Ankara City Hospital, Ankara, Turkey; 4 Faculty of Medicine, Department of Pediatric Nephrology, Adana Başkent University, Adana, Turkey; 5 Department of Microbiology, Ankara Gülhane Training and Research Hospital, Ankara, Turkey; 6 Department of Pediatric Nephrology, University of Health Sciences, Ankara City Hospital, Ankara, Turkey; 7 Department of Pediatric Endocrinology, University of Health Sciences, Adana City Hospital, Adana, Turkey; 8 Faculty of Medicine, Department of Pediatric Endocrinology, Çukurova University, Adana, Turkey

**Keywords:** Plasminogen, Plasminuria, Children, Type 1 diabetes mellitus, Diabetic kidney disease, Diabetic nephropathy

## Abstract

This study aims to determine the role of urinary plasminogen, a serine protease that reflects damage to the glomerular filtration barrier, in the early detection of diabetic kidney disease in pediatric patients with type 1 diabetes mellitus (T1DM). Pediatric patients, who had been followed up with T1DM for at least 2 years, and healthy matched controls were included. The association between urine plasminogen (uPlasminogen) levels and urine plasminogen-to-creatinine ratio (uPlgCR) with urine albumin-to-creatinine ratio (uACR) was examined, as well as determining a cut-off value for uPlasminogen to predict microalbuminuria. A total of 56 T1DM patients and 30 healthy matched controls were included. There was no significant difference in eGFR (estimated glomerular filtration rate) and systolic and diastolic blood pressure between the groups. uPlasminogen level was determined to be higher in the albuminuric group than control (*p* = 0.001) and uPlgCR was higher in the normoalbuminuric and albuminuric groups than control (*p* = 0.004, *p* = 0.002, respectively). There was a weak positive correlation between uPlasminogen and uPlgCR with uACR (*r* 0.38,* p* = 0.004; *r* 0.28, *p* = 0.03, respectively). The diagnostic performance of uPlasminogen in predicting albuminuria was evaluated by ROC-curve analysis, showing an AUC of 0.69 (*p* = 0.02; 95% CI [0.521–0.861]), with a sensitivity of 75% and specificity of 65.5% at a cut-off value of 7.1 µg/L. *Conclusion: *A positive correlation was found between uPlasminogen and uPlgCR with albuminuria. Also, uPlasminogen was determined to predict albuminuria. Most importantly, uPlgCR was elevated in diabetic patients before albuminuria developed. uPlgCR may serve as a potential early marker or therapeutic target of DKD, pending further longitudinal validation.

**Table:** 

**What is Known:** • *Urinary plasminogen correlates with albuminuria in diabetic patients.*	
**What is New:** • *Urinary plasminogen and uPlgCR levels exhibited a significant and positive correlation with uACR. The new findings in this study are that uPlgCR was detected in urine before microalbuminuria develops, and that uPlasminogen predicted albuminuria in pediatric patients with T1DM.*

**Supplementary information:**

The online version contains supplementary material available at 10.1007/s00431-025-06278-3.

## Introduction

T1DM is a common chronic disease among children, and DKD is one of the most common and important microvascular complications of T1DM [1]. The morphological and functional alterations in the kidney among patients with T1DM are generally classified into five stages. The first stage is characterized by hyperfiltration, glomerular hypertrophy, and hyperperfusion, which are the earliest changes. In stage 2, there is subclinical morphologic alteration with increased albumin excretion rates within the normal range. Overt microalbuminuria is found in the third stage and macroalbuminuria in the fourth stage, which generally occurs 10–15 years after the onset of T1DM. Kidney failure is the fifth and last stage of DKD [2]. Although advanced stages of DKD are rare among children and adolescents with T1DM, early renal changes develop soon after diagnosis of diabetes [2].

Microalbuminuria is an important and early sign of DKD, and the mortality of DKD is significantly higher with elevated albuminuria [1]. Recent studies reported that albuminuria can also be reversible [2]. A high percentage of regression to normo-albuminuria in children having a history of albuminuria raises the question if albuminuria is a valid early indicator of DKD. Even though urinary albumin is monitored clinically as a first marker of DKD, new biomarkers are being investigated to detect DKD early since regression to normo-albuminuria may occur [3]. It was demonstrated that additional bloodstream proteins pass through damaged glomeruli and are detected in the urine [4], including the thrombolytic protease plasmin and its precursor plasminogen [5]. Plasmin and plasminogen can be detected in the urine of patients with glomerular disease [6]. Plasminogen is one of the urinary serine proteases that may contribute to renal Na^+^ retention by activating the epithelial Na^+^ channel (ENaC). Observations indicating the association of urinary levels of plasmin and plasminogen with elevated extracellular fluid volume suggest a role for plasmin in Na^+^ retention [5, 7].

During glomerular diseases, plasminogen can be filtered aberrantly and pass into the tubular space [5, 8, 9]. Therefore, it is expected that urinary excretion of plasminogen parallels albuminuria. There are many experimental studies evaluating the role of urinary plasminogen in glomerular diseases in the literature, but the number of clinical studies is limited, particularly in pediatric patients [10–12]. Plasminogen is thought to be increased in the urine as an early sign of renal damage before microalbuminuria occurs in patients diagnosed with pediatric diabetes mellitus, and this can also be a therapeutic target to slow disease progression.

For this purpose, the presence of plasminogen was examined in the urine of pediatric patients with T1DM and healthy controls. Besides, the urinary plasminogen levels were also assessed in relation to the presence of albuminuria in pediatric T1DM patients. Detection of plasminuria in the early stages may be a marker that can be used in the clinical setting and will also constitute a target for future treatments.

## Materials and methods

Children under 18 years old diagnosed with T1DM for at least 2 years, who were followed up in our pediatric endocrinology department, were included in this study. All patients were under an intensive insulin regimen (glargine and rapid insulin). This study was carried out between April 2019 and January 2021 and was conducted at Ankara Child Health Hematology-Oncology Training and Research Hospital. The patients who had any other glomerular disease and urinary tract anomalies and those with a history of intense exercise, menstrual bleeding, any febrile infection, urinary tract infection, significant hyperglycemia at the time of investigation, and smokers were excluded. Age- and gender-matched healthy children were involved as the control. The controls were recruited among the children who were referred to the pediatric outpatient clinics for routine follow-up. The control participants had no underlying acute or chronic disease and were not taking any medications. Kidney functions, urine analysis, and blood pressure of controls were all normal. Controls with a history of intense exercise, menstrual bleeding, febrile infection, and smoking were also excluded. Both patients and healthy controls were enrolled in this study consecutively during the same period. A flowchart summarizing patient and control enrollment and exclusion criteria is presented in Supplementary Online Fig. 1 and 2.

Informed consent was obtained from both patients and healthy children and their parents. Participants’ birth dates, date of diabetes diagnosis, duration of diabetes, gender, height, and weight measurements, and BMI (kg/m^2^) values were recorded. Body mass index percentile was calculated using an online calculator (https://www.childmetrics.org) [13]. After 5-min resting, blood pressure measurement was performed in a seated position by using a validated automated oscillometric device (OMRON M2 Basic, HEM-7121-E, Omron Healthcare). Three measurements were performed at 15-min intervals, and the average value was calculated.

Fasting morning blood and morning first urine samples were obtained from all participants. Plasma creatinine, albumin, electrolytes, glycosylated hemoglobin (HbA1c), and urinary albumin and creatinine were analyzed. Urine samples were collected in 50-mL sterile tubes. After centrifugation of urine for 20 min at 1000 g at room temperature, the supernatant was collected and kept at − 80 °C before the analysis of plasminogen. The plasminogen concentration was evaluated using the ELISA method (Cloud-Clone Corp. 23,603 W. Fernhurst Dr. Unit 2201, Katy, TX77494, USA). The ELISA process was conducted by following the manufacturer’s instructions [14]. Intra-assay precision was assessed by measuring three samples with low, medium, and high concentrations of plasminogen 20 times on a single plate. Inter-assay precision was evaluated by testing the same three concentration levels across three independent plates, with eight replicates per plate. The coefficient of variation (CV%) was calculated utilizing the formula (standard deviation/mean) × 100. The intra-assay CV was found to be < 10% and the inter-assay CV to be < 12%. Urinary creatinine was measured by using the spectrophotometric method and a commercial kit (Siemens, Advia 1800 Chemistry System, Germany). Plasmin(ogen) levels were normalized to urinary creatinine to adjust for differences in urine concentration among participants. HbA1c was measured by using the high-pressure liquid chromatography method with a commercial kit (BioRad D-10 Hemoglobin Testing System, France).

Estimated glomerular filtration (eGFR) rates were computed employing the modified Schwartz formula [15]. Spot uACR < 30 mg/g was classified as normo-albuminuria, > 30 mg/g as albuminuria (30–299 mg/g: microalbuminuria, and > 300 mg/g as macroalbuminuria [16]. The patient group was divided into two subgroups (normoalbuminuric and albuminuric) by considering the uACR. If the patients had albuminuria in two or all of three urine samples during the last 6 months of follow-up, these patients were included in the albuminuric group; others were in the normoalbuminuric group [2].

### Statistical analysis

Statistical analyses were conducted using the IBM SPSS version 22.0. Qualitative variables were compared utilizing the chi-square test and presented as numbers and percentages (%). They were tested for normality by employing the Kolmogorov–Smirnov test. Normally distributed ones were presented as mean and standard deviation (SD). Non-normally distributed ones were presented as median and interquartile range (IQR). Two non-normally distributed quantitative variables were compared employing the Mann–Whitney *U* test, and three groups were compared by using the Kruskal–Wallis test. One-way ANOVA test was employed in comparing three groups, with the Bonferroni post hoc test being used for pair-wise comparison (type 1 error: alpha/3 = 0.0167). Spearman correlation was employed for non-parametric correlations between quantitative variables and Pearson correlation for parametric correlations (*r* = 0.10–0.39, weak correlation; *r* = 0.40–0.59, moderate correlation; *r* = 0.6–0.79, strong correlation). ROC curve was utilized to assess the diagnostic value of uPlasminogen and uPlgCR in the prediction of albuminuria in children with T1DM. AUC, specificity, sensitivity, accuracy rate, and positive and negative predictive values were determined considering the ROC. The best cut-off points with relevant specificity and sensitivity were defined by using the Youden Index. Additionally, univariate and multivariate Logistic Regression analyses were employed to reveal the factors affecting albuminuria. Variables that are not diagnostic for diabetes but may predispose albuminuria were selected as predictive variables. Data regarding age of diabetes diagnosis, time of diabetes duration, and HgbA1c values did not exist for control group so we did not include these parameters for univariate and multivariate logistic regression analysis. Statistical significance was set at *p* < 0.05.

## Results

Fifty-six patients diagnosed with T1DM and 30 healthy controls were involved in this study. Demographic, clinical, and laboratory data of normoalbuminuric, albuminuric T1DM, and control groups are compared in Table 1. Only 1 patient had macroalbuminuria in the albuminuric group with T1DM. The groups were homogeneous in terms of age, gender, BMI, systolic and diastolic blood pressure, and eGFR. Normoalbuminuric and albuminuric patients with T1DM were also compared among themselves, and they were found to be homogenous regarding age of diabetes diagnosis, duration of diabetes, and HbA1c levels. Considering the duration of diabetes, 21.4% of the patients had been followed up for 2–5 years, 58.9% for 6–10 years, and 19.6% for more than 10 years.

**Table 1:** Demographic, clinical, and laboratory results of the T1DM and control groups

	Normoalbuminuric T1DM (*n* = 40)	Albuminuric T1DM (*n* = 16)	Control **(***n*** = **30)	*p* value
Sex (F/M)	15/25	9/7	12/18	0.428
	Mean ± SD Median (IQR)	Mean ± SD Median (IQR)	Mean ± SD Median (IQR)	
Age (year)	13.0 ± 3.6 13.1 (5.7)	14.2 ± 2.9 15.0 (5.2)	13.6 ± 3.4 13.5 (5.8)	0.617
Age of diabetes diagnosis (years)	5.6 ± 3.3 5.4 (5.3)	7.1 ± 3.6 6.7 (4.3)		0.211
Time of diabetes duration (months)	89.05 ± 36.1 89.5 (42.0)	86.11 ± 42.5 83.2 (62.8)		0.786
BMI percentile (%)	51.1 ± 32.5 52.9 (66.9)	56.7 ± 25.3 58.2 (41.9)	51.2 ± 33.3 54.8 (65.7)	0.844
Mean systolic blood pressure (mmHg)	109.3 ± 10.2 111.5 (18.7)	106.8 ± 12.9 107.5 (24.5)	107.4 ± 11.7 107.5 (21.7)	0.591
Mean diastolic blood pressure (mmHg)	62.9 ± 5.9 63.5 (8.7)	61.1 ± 7.1 60.5 (13.2)	61.6 ± 6.3 61.0 (9.2)	0.541
HbA1c (%)	8.8 ± 1.7 8.4 (2.0)	8.7 ± 1.5 8.2 (2.0)		0.978
eGFR (ml/dk/1.73 m^2^)	91.8 ± 12.0 89.4 (58.5)	88.6 ± 9.9 85.4 (13.5)	91.3 ± 11.4 92.5 (13.8)	0.437
Spot urine albumin/creatinine (uACR) (mg/g)	8.4 ± 6.0 6.6 (5.3)	123.4 ± 152.1 50.1 (156.8)	5.8 ± 2.9 6.0 (5.0)	** < 0.001**
Spot urine plasmin(ogen) (µg/L)	7.7 ± 5.8 5.8 (6.1)	12.7 ± 7.2 11.6 (14.4)	5.03 ± 2.5 3.9 (2.7)	**0.002**
Spot urine plasmin(ogen)/creatinine (uPlgCR) (µg/g)	14.1 ± 16.9 6.9 (12.4)	18.1 ± 15.6 16.0 (22.2)	5.4 ± 4.9 3.9 (4.5)	**0.001**

*BMI*; body mass index, *HbA1C*; glycated hemoglobin, *uACR*; urinary albumin/creatinine ratio, *uPlgCR*; urinary plasmin(ogen)/creatinine ratio

**Fig. 1:**
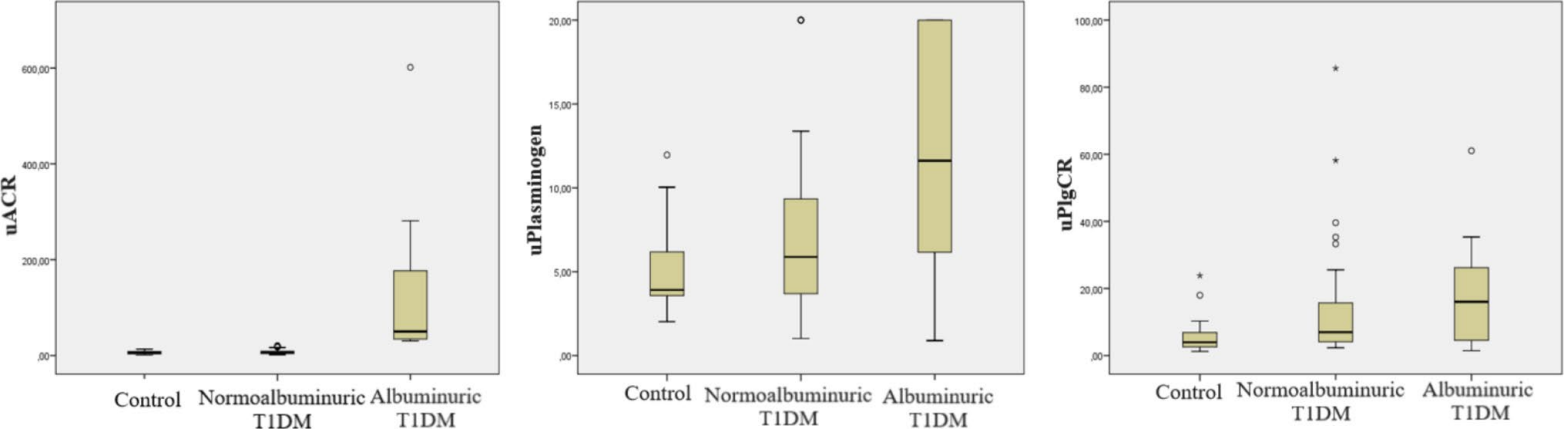
uACR, uPlasminogen, and uPlgCR levels by groups. Abbreviations: uACR (mg/g), urinary albumin/creatinine ratio; uPlasminogen (µg/L), urinary plasminogen; uPlgCR (µg/g), urinary plasminogen/creatinine ratio

The uPlasminogen level was higher in the albuminuric group than the control (*p* = 0.001), with no significant difference between the normoalbuminuric and control groups and between normoalbuminuric and albuminuric groups. The uPlgCR ratio was higher in both normoalbuminuric and albuminuric groups than the control (*p* = 0.004 and *p* = 0.002, respectively) (Fig. 1).

No statistically significant correlation was found between uPlasminogen and diabetes duration time, BMI, eGFR, and HbA1c level in this cohort, but a weak positive correlation was determined between uPlasminogen and uACR (*r* 0.38, *p* = 0.004). Similarly, no statistically significant correlation was found between uPlgCR and diabetes duration time, BMI, eGFR, and HbA1c level in this cohort, but a weak positive correlation was detected between uPlgCR and uACR (*r* 0.28,* p* = 0.03).

**Fig. 2:**
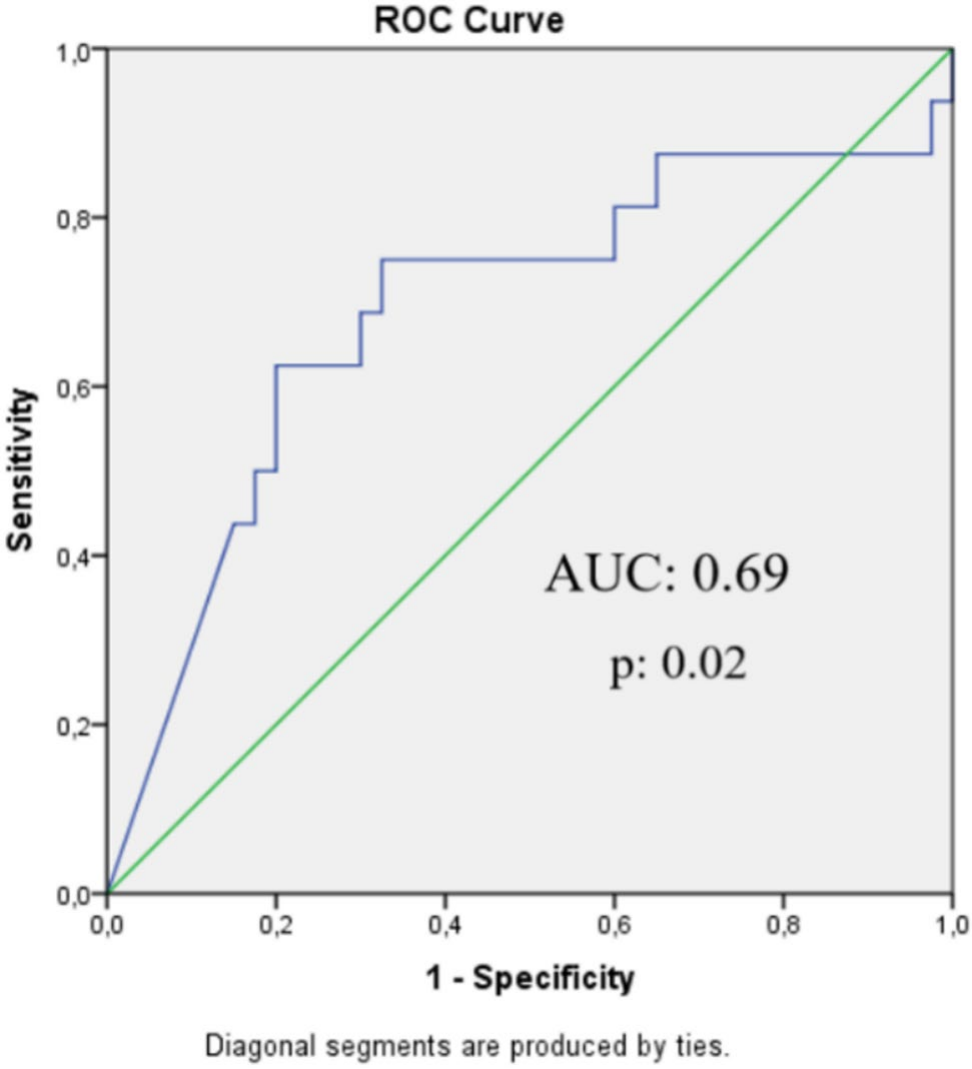
ROC curve of uPlasminogen in the prediction of albuminuria. Abbreviations: AUC, area under the curve

The diagnostic value of uPlasminogen was analyzed with ROC curve (Fig. 2), and the results revealed diagnostic accuracy of uPlasminogen in prediction of albuminuria in T1DM children (AUC:0.69, *p* = 0.02; 95% CI [0.521–0.861]), but the uPlgCR was not diagnostic in prediction of albuminuria (*p* 0.258, ROC curve). The best cutoff value for uPlasminogen to predict albuminuria was found to be 7.1 µg/L (specificity, 65.5%; sensitivity, 75%) (Table 2).

**Table 2:** Assessment of the uPlasminogen in the prediction of albuminuria in children with T1DM

	AUC	Cut-off point	Sensitivity (%)	Specificity (%)	PPV (%)	NPV (%)	Accuracy (%)	95% CI
uPlasminogen	0.69 (*p*:0.02)*	**7.1µg/L**	75	65.5	48	87.1	69.6	0.521–0.861

*AUC*; area under the curve, *PPV*; positive predictive value, *NPV*; negative predictive value, *CI*; confidence interval

*p < 0.05 indicates that the AUC is significantly different from random chance

Examining the factors affecting albuminuria by employing both univariate and multivariate logistic regression analyses, uPlasminogen > 7.1 µg/L was determined to increase the risk of albuminuria by 12 times, and other variables were found to have no significant effect. In addition, uPlasminogen was shown to have an independent effect on albuminuria and did not interact with age, sex, BMI percentile, and mean SBP (Table 3).

**Table 3:** Factors affecting albuminuria in univariate and multivariate logistic regression, including interaction terms with uPlasminogen

Univariate analysis	*B*	SH(B)	OR	95% for OR	*p*
uPlasminogen > 7.1 µg/L	**2.485**	**0.736**	**12.00**	**2.836–50.775**	0.001*
Age	0.068	0.099	1.071	0.882–1.299	0.489
Sex (female)	0.657	0.627	1.929	0.565–6.588	0.295
BMI percentile	0.006	0.010	1.006	0.986–1.027	0.560
Mean SBP	− 0.004	0.026	0.996	0.947–1.048	0.887
Multivariate analysis	*B*	SH(B)	OR	95% for OR	*p*
uPlasminogen > 7.1 µg/L	**2.458**	**0.770**	**11.682**	**2.582–52.865**	0.001*
Age	− 3.196	4.201	0.041	0.774–1.315	0.447
Sex (female)	0.321	0.814	1.378	0.279–6.797	0.694
BMI percentile	0.005	0.015	1.005	0.976–1.034	0.741
Mean SBP	0.010	0.051	1.010	0.914–1.117	0.839
Interaction terms with uPlasminogen	*B*	SH(B)	OR	95% for OR	*p*
Age	− 0.066	0.243	0.936	0.581–1.508	0.787
Sex (female)	− 0.174	1.479	0.840	0.046–15.246	0.906
BMI percentile	0.025	0.027	1.026	0.573–1.081	0.375
Mean SBP	− 0.150	0.078	0.861	0.738–1.003	0.055

Interaction terms evaluate whether the effect of uPlasminogen > 7.1 µg/L on albuminuria is modified by age, sex, BMI percentile, or mean systolic blood pressure (SBP). None of the interaction terms were statistically significant

*BMI*; body mass index, *SBP*; systolic blood pressure, *OR*; odds ratio

*p: Logistic regression

## Discussion

This study aims to evaluate the role of uPlasminogen and uPlgCR to detect DKD early. In clinical practice, albuminuria is the most significant marker for the prediction and detection of DKD. Early diagnosis and treatment of DKD prevent progression to end-stage renal failure in children with T1DM [2]. Plasminogen, one of the urinary serine proteases known to increase in urine as a result of damage to the glomerular filtration barrier, is correlated with microalbuminuria or macroalbuminuria in DM [5, 9]. Similar observations were made in patients with pre-eclampsia and nephrotic syndrome [8, 17]. This study revealed that the uPlasminogen and uPlgCR levels were significantly and positively correlated with uACR and, more importantly, verified that uPlgCR was detected in urine before the development of microalbuminuria in patients with T1DM. Considering these results, it is suggested that uPlasminogen and uPlgCR may be used as markers for DKD and uPlgCR as an early biomarker for DKD.

The plasminogen, with a molecular weight of 92 kDa, is heavier than albumin [18]. The fact that it is seen in the urine before albuminuria in patients with T1DM creates a contradiction in the understanding that it is a result of glomerular damage. A variety of mechanisms, ranging from glomerular damage to tubular changes, play a role in the pathophysiology of DKD [19]. Renal tubular changes, especially in the proximal tubule, gained increasing significance and provide new insight into the understanding of early-stage DKD [20]. In particular, there is increasing evidence for the importance of urinary proteins other than albumin as markers in the early stages [19]. The proximal tubule is at a high risk of exposure to hypoxia, especially in diabetic conditions [21]. The study carried out by Roelofs J.J.T.H et al. [22] reported a prominent increase in tissue-type plasminogen activator (tPA) expression in damaged renal tubules during ischemia. In addition to passage through damaged glomeruli, active plasminogen may increase in the tubular lumen regardless of molecular weight in patients with DKD.

Classical history of DKD was described as an initial rise in urine albumin excretion, then progressive decrease in glomerular filtration rate, and finally end-stage renal disease, which develops several decades after initial diagnosis of DM [1, 2]. Recent studies refined the natural history of DKD and reported that kidney biopsies performed 1.5 to 5 years after the initial diagnosis of diabetes show the characteristic structural changes of DKD in children [23–25]. In the present study, the duration of diabetes is not as long as in adult studies, and the duration of diabetes does not differ between the normoalbuminuric and albuminuric groups. This finding suggests that there may be factors other than the duration of diabetes affecting diabetic kidney disease. Poorly controlled diabetes and high blood pressure may contribute to the early occurrence of DKD. In this study, HbA1c and systolic and diastolic blood pressure values did not differ in the normoalbuminuric and albuminuric groups. Further studies are needed for other reasons.

Even though the current clinical hallmark for diagnosis of DKD is still albuminuria, the histopathological changes related to advanced stages of DKD may precede the onset of albuminuria [25]. Recent studies also demonstrated that patients with both T1DM and type 2 DM (T2DM) may present with renal dysfunction without having proteinuria, described as nonproteinuric nephropathy [26]. Considering these new developments in DKD, less emphasis should be placed on albuminuria and more to the mechanism that initiates and supports progression to chronic kidney disease [27]. In this context, uPlasminogen and uPlgCR were reported to be remarkable biomarkers in DKD [5]. Buhl et al. [9] showed that uPlgCR levels were significantly elevated in both microalbuminuric and macroalbuminuric patients with T2DM when compared with normoalbuminuric patients. They also detected that uPlgCR levels were correlated significantly with urinary albumin. Andersen et al. [5] reported that macroalbuminuric T1DM patients had significantly higher levels of uPlgCR than normoalbuminuric T1DM patients (172 µg/g and 0.348 µg/g, respectively). In line with these studies, the present study revealed that uPlasminogen and uPlgCR increased in pediatric T1DM patients who had albuminuria. Additionally, it was also shown that uPlgCR was higher in normoalbuminuric T1DM patients compared to healthy controls. The results achieved in this study are particularly important because, reviewing the literature, no pediatric studies comparing diabetic patients with healthy controls in order to detect plasminogenuria could be found.

It was also aimed to establish a cutoff value for uPlasminogen, and it was found that a urinary level of 7.1 µg/L will predict albuminuria in diabetic patients. Additionally, it was determined that uPlasminogen > 7.1 µg/L increased the risk of albuminuria by 12 times. Reviewing the literature, no study indicating that plasminuria predicts albuminuria in patients with T1DM could be found. Given the results achieved in this study, it is suggested that in normoalbuminuric diabetic patients with uPlasminogen levels of ≥ 7.1 µg/L, urine albumin levels should be monitored closely, and glycemic control should be improved in order to prevent progression of glomerular damage.

Microalbuminuria has been considered a strong predictor of progression to end-stage kidney disease [27]. However, microalbuminuria as a diagnostic tool for the onset of DKD has its shortcomings. Perkins et al. [27] reported development of advanced chronic kidney disease in one-third of T1DM patients relatively soon after the onset of microalbuminuria, and this was not conditional on the presence of proteinuria. Continuous exposure to excessive transglomerular passage of plasminogen in glomerular injury acts as a “second hit” to glomerular disease, causing progression of chronic kidney disease independent of the initial injury [28]. In this context, plasminuria may not only be a disease marker in pediatric patients with T1DM but may also be used to predict disease progression [29]. This can be examined in further studies and long-term follow-ups. Considering the findings achieved in the present study, plasminogen may be a marker for renal damage in non-albuminuric DKD, but more studies are needed on its contribution to disease progression and mortality.

Plasminogen is abnormally filtered across the glomerular barrier in proteinuria and is activated in the tubular fluid, where plasmin can proteolytically activate the ENaC [30]. The activation of ENaC in microalbuminuric conditions could contribute to hypertension [9]. Considering the results reported in previous studies, it is thought that plasminuria is not only a disease marker but also a risk factor for kidney damage due to its effect on sodium balance. Buhl et al. [9] showed that uPlgCR predicted the daytime systolic blood pressure, and they also suggested that urinary plasmin activity could predict cardiovascular outcome in diabetic patients. In the present study, no difference was found in mean systolic and diastolic blood pressures between the groups. Among the patients involved in this study, only the patient with macroalbuminuria was receiving enalapril, whereas other patients were not on any antihypertensive medication. The fact that most patients were in the normoalbuminuric group (71.4%), as well as the short diabetes duration, might have affected these results.

In a study on nephrotic puromycin aminonucleoside rat model, amiloride reduced urinary protease activity and plasmin/plasminogen ratio [10]. This suggests that urinary plasmin activity is a target for amiloride. The urinary plasmin activity may be a target for new treatments in early diabetic nephropathic changes, where microalbuminuria has not yet occurred. However, more studies are needed to determine whether urinary plasminogen could be a treatment target in DKD.

Given the small sample size, this study has several limitations. The average duration of diabetes was less than 10 years in each diabetes group, and therefore, the rate of patients in the albuminuric groups was less than the rate of patients in the normoalbuminuric group (28.5%, 71.4% retrospectively). Early increases in uACR, even within the normal range, can occur during the first years after diagnosis and can predict future albuminuria risk. It was recommended that albuminuria screening in children with T1DM be initiated at 2–5 years of diabetes duration [2]. One of the limitations is the weak AUC value of ROC analysis for uPlasminogen in predicting albuminuria. The ROC AUC value ranges between 0 and 1, with 0.5 indicating random guessing and 1 indicating perfect performance for predicting the disease. The weak AUC value is one of the limitations of this study. Moreover, the sensitivity was found to be 75%, and the small sample size might have caused this result. More studies with more cases and involving multicentric and longitudinal observation of the disease course are needed to validate the results. Another limitation of this study is the lack of ambulatory blood pressure monitoring (ABPM). Blood pressure increases as a result of activation of ENaC by plasmin in patients with diabetes. Conducting multiple office blood pressure measurements may be an option for diagnosing hypertension, but annual ABPM is recommended in pediatric diabetic patients [31, 32].

In conclusion, children with T1DM are at risk for developing microvascular complications such as diabetic kidney disease. Early detection of T1DM complications is important to prevent long-term morbidity and mortality. Moreover, early diagnosis and prompt treatment of DKD prevent progression to end-stage kidney disease in children with T1DM. This study demonstrated that uPlasminogen and uPlgCR correlate with uACR and that uPlasminogen could predict albuminuria in diabetic patients. In addition, uPlgCR was elevated in diabetic patients before the development of albuminuria. Even though larger and longitudinal studies are needed for validation, uPlgCR may serve as a potential early marker of DKD or therapeutic target for new treatments.

## Supplementary information

Below is the link to the electronic supplementary material.

ESM 1 (DOCX 35.0 KB)

ESM 2 (DOCX 33.1 KB)

## Data Availability

No datasets were generated or analysed during the current study.

## Abbreviations

ABPM: Ambulatory blood pressure monitoring
DM: Diabetes mellitus
DKD: Diabetic kidney disease
eGFR: Estimated glomerular filtration rate
ENaC: Epithelial Na^+^ channel
T1DM: Type 1 diabetes mellitus
T2DM: Type 2 diabetes mellitus
uPlgCR: Urinary plasminogen to creatinine ratio
uACR: Urinary albumin to creatinine ratio
uPlasminogen: Urinary plasminogen
