# Supplementary material for: Urinary plasminogen as an early marker of diabetic kidney disease in children with type 1 diabetes mellitus: a cross-sectional study
Source: Eur J Pediatr. 2025 Jun 27;184(7):451. doi: 10.1007/s00431-025-06278-3 (PMC12204940; doi:10.1007/s00431-025-06278-3)
Supplement: Supplementary file 1 — (DOCX 35.0 KB) [file 431_2025_6278_MOESM1_ESM.docx]

Supplementary Online Figure 1. A flowchart summarizing patient enrollment and exclusion criteria

Abbreviations: T1DM: Type 1 diabetes mellitus; T2DM: Type 2 diabetes mellitus; MODY: maturity onset diabetes of young; uACR: Urine albumin creatinine ratio

uACR ≥ 30 mg/g

Albuminuric

(n=16)

uACR < 30 mg/g

Normoalbuminuric

(n=40)

56 patients with T1DM included

11 patients refused to participate in the study

1 patient dropped out

15 patients were excluded

Other glomerular disease

Diabetic ketoacidosis

Urinary tract anomalies

Urinary tract infections

Other febril infections

Intensive exercise

Menstruel bleeding

Smoking

68 patients with T1DM were enrolled

83 patients with T1DM were selected

34 patients were excluded

T2DM, MODY

>18 years old

Following <2 years

117 patients followed up in the diabetes outpatient clinic of the pediatric endocrinology department
