# Supplementary material for: Urinary plasminogen as an early marker of diabetic kidney disease in children with type 1 diabetes mellitus: a cross-sectional study
Source: Eur J Pediatr. 2025 Jun 27;184(7):451. doi: 10.1007/s00431-025-06278-3 (PMC12204940; doi:10.1007/s00431-025-06278-3)
Supplement: Supplementary file 2 — (DOCX 33.1 KB) [file 431_2025_6278_MOESM2_ESM.docx]

Supplementary Online Figure 2. A flowchart summarizing controls enrollment and exclusion criteria

13 patients were excluded

Urinary tract anomalies

Urinary tract infections

Other infections

Intensive exercise

Menstruel bleeding

Smoking

30 patient included to control group

35 patients were excluded

24 patients refused to participate in the study

54 patients were enrolled

67 patients were enrolled

**Inclusion criteria**

<18 years old

No known chronic disease

No medications

Normal kidney function

Normal urine analysis

Normal blood pressure

102 patients applied for routine check-up without any complaints

1127 patients were admitted to the pediatric outpatient clinic
